# Development of Immune Cells in the Intestinal Mucosa Can Be Affected by Intensive and Extensive Farm Environments, and Antibiotic Use

**DOI:** 10.3389/fimmu.2018.01061

**Published:** 2018-05-16

**Authors:** Zoe Christoforidou, Rachel Burt, Imke Mulder, Bhupinder P. Gill, John Pluske, Denise Kelly, Christopher R. Stokes, Michael Bailey, Marie C. Lewis

**Affiliations:** ^1^Infection and Immunity, School of Veterinary Science, University of Bristol, Langford, United Kingdom; ^2^Gut Immunology Group, Rowett Institute, University of Aberdeen, Aberdeen, United Kingdom; ^3^Agricultural and Horticultural Development Board, Milton Keynes, United Kingdom; ^4^School of Veterinary and Life Sciences, Murdoch University, Murdoch, WA, Australia; ^5^Food and Nutritional Sciences, School of Chemistry, Food and Pharmacy, University of Reading, Reading, United Kingdom

**Keywords:** immune development, neonate, regulatory T-cells, dendritic cells, farm environment, antigen presentation, mucosal immunology

## Abstract

Epidemiological studies have demonstrated that exposure to farm environments during childhood can be linked to reductions in the incidence of immune disorders, but generating an appropriate model is difficult. 108 half-sibling piglets were born on either extensive (outdoor) or intensive (indoor) farms: at 1 day old, a subset of piglets from each litter were transferred to a high-hygiene isolator facility to create differences in rearing environment either during birth/first day or during the subsequent 56 days of life. Interactions between CD14, CD16, MHCIIDR, and capillary endothelium were assessed using four-color quantitative fluorescence immunohistology. Effects of birth and rearing environment on the antigen-presenting microenvironment of the proximal and distal jejunum (professional and stromal) were apparent at 5, 28, and 56 days after birth However, effects on CD4^+^CD25^+^Foxp3^+^ regulatory T-cells (T_regs_) in the intestinal mucosa were apparent around weaning at 28 days but had disappeared by 56 days. These T_regs_ were reduced in the isolator piglets compared to their farm-reared siblings, but this effect was less marked in piglets born on the extensive farm and required administration of antibiotics. Our results suggest that there may be at least two windows of opportunity in which different farm environments were influencing immune development: one during the perinatal period (up to the first day of life), and one during later infancy. Furthermore, the differences on T_regs_ suggest that the effects of early life influences may be particularly critical around weaning.

## Introduction

Non-communicable inflammatory, autoimmune, and allergic diseases represent an increasing challenge for twenty-first century medicine. Although many have genetic components, environmental triggers appear important, and can originate during early-life “programming events” (1, 2). This term refers to critical points of developmental plasticity where environmental factors can have significant implications for long-term health (3, 4). There is now considerable evidence that different rearing conditions, and antibiotic treatment in early in life, affect the development of the immune system, probably by modifying microbial stimuli at the gut and other mucosal surfaces (5–9). Disruption in development of the microbiota is being increasingly linked to later immunological disorders, including asthma and other allergies (10, 11), and acquisition of this early microbiota is highly dependent on neonatal and childhood environments (12), as well as maternal influences. Our earlier work indicates that the pattern of acquisition of intestinal bacteria during the first day of life can be modified by relatively minor alterations in environmental conditions, including the farm of origin and rearing environment, thus altering its developmental trajectory, and that of the associated immune system (13–16).

Children raised in farm environments have a lower prevalence of allergic disease than children in urban areas (17, 18), and the difference is greatest among children of full-time compared to part-time farmers (19). Farm environments, in addition to increased exposure to animals, may include the consumption of non-pasteurized milk and dairy farm exposure during the first year of life confers the greatest protective impact compared to other farming practices (20). In addition, farm dwelling often means living in less “hygienic” conditions, such that infants are exposed to a greater variety of novel antigens than their urban-dwelling counterparts. A recent study found a significant decrease in asthma and other allergies in the children of Amish compared to Hutterite farming communities (21). Interestingly, this study accounted for other factors known to influence allergy development, including family size, contact with pets, vaccine uptake, genetic ancestry, childhood obesity, smoking, extended breastfeeding, and diet (including raw milk consumption). This observed difference appeared to be driven by the type of farm exposure: while the Amish were traditional farmers, used horses, and were exposed to a more varied microbial content in house dust, the Hutterite used a more industrialized approach. It is unlikely that the protective qualities of the farm environment, in terms of immunological disorders, will be reduced to a single factor but instead attributable to a plethora of antigen exposures which have not yet been characterized.

There is also clear evidence for the link between early life antibiotic administration and development of allergies later in life (5, 22, 23). Antibiotic usage and allergy studies need to be interpreted with care, since it may be difficult to distinguish between actual respiratory infection in children and the first symptoms of asthma, such that early-life antibiotic administration may be a consequence of asthma rather than a cause (24). However, correlation between antibiotics’ exposure before 6 months and asthma diagnosis after 3 years of age (25) reduces the likelihood that these early symptoms were asthma related. Furthermore, the effect of antibiotics on asthma development was stronger in non-genetically predisposed children, suggesting that this may be the reason for the apparent absence of an effect in studies where only genetically at-risk individuals were assessed. Together, these findings are consistent with different early life environments affecting the development of the microbiota, and may also affect how the immune system develops and responds to different antigens in later life.

Here, we focus on two aspects of the developing mucosal immune system: the cells in the intestinal lamina propria involved in presentation of antigen to CD4^+^ T-cells, and the types of CD4^+^ T-cell (effector or regulatory) which accumulate in those tissues. Our preliminary work has provided direct evidence that living in a farm environment for the first 28 days of life correlates with an increase in the number of CD4^+^Foxp3^+^ regulatory T-cells (T_regs_) in the gut mucosa as determined by quantitative immunohistology. This finding occurred alongside a significant decrease in the area staining positive for CD4 in the lamina propria of the distal jejunum, which presumably reflects the CD4^+^ T-cells with the potential for memory/effector function. This resulted in a significantly increased proportion of T_regs_ within CD4 T-cells in the early gut mucosa, and thus, an increased potential for long-term immune tolerance. Consistent with this, the increased proportion of T_regs_ within CD4 T-cells was linked to a reduction in the specific systemic response to novel food antigens introduced at weaning, indicating a functional consequence (26). However, the mechanisms linking early-life environment to later development of allergy are still unclear. Diet and the gut microbiota have been shown to affect the number and function of T_regs_ in both the intestinal mucosa and periphery in adult mice (27). However, manipulating the neonatal environment of rodents in a manner relevant to human infants is demanding.

Antigen presentation is a pivotal factor in the development of a well-functioning immune system and the gut, in particular, is a critical site for initiating antigen presentation since it is the largest interface between the host and its environment, and because of the large antigen load and microbiota. Dendritic cells (DCs) from the intestine clearly do recirculate to regional lymph nodes like the mesenteric nodes, and presentation in these sites can polarize both T-cell subset differentiation and homing (28, 29). However, it is well established that DCs also express MHCII while migrating through peripheral tissues and, more recently, primed T-cells have been demonstrated to switch polarization status long after exiting the lymph nodes (30). Current evidence suggests that antigen presentation outside of these sites, by conventional and stromal antigen-presenting cells (APCs), may also be important in directing divergent immune responses in humans, pigs, and rodents (31).

Classification of DC subsets is dependent on function, expression of cell surface molecules and, recently, analysis of high-dimensional flow cytometry data or of gene expression by single, sorted cells (32, 33). However, the extent to which these findings are applicable across all mammals is unclear. Although SIRPα (CD172a) and CD1 have been used to characterize blood DCs in the pig, we and others have previously shown that functionally active, CD4^−^ DCs within the intestinal mucosa of pigs fall into two categories, characterized by expression of the molecules FcγRIII (CD16), CD172a, and the integrin alpha chain CD11R1 (31, 34, 35). Interestingly, expression of CD16 has been shown to accurately delineate the CD141^−^, CD1^−^ subset of circulating human DCs (33), and in pigs, expression of CD16 is also limited to the CD1^−^ subset of circulating DCs (36). The CD16^+^ subset of intestinal DCs is identifiable *in situ* by immunohistology (31) and recruitment to the intestine appears to be modulated by early life microbiome (37). In addition, we have previously shown that MHCII^+^ endothelium is capable of functional presentation of antigen and that it co-localizes with professional APCs and CD4^+^ T-cells in neonatal piglets but not in adults, consistent with antigen presentation by the endothelium playing an important role in the development of the immune system in the young of some species (31). It follows that changes in microbial exposure due to neonatal and rearing environments may influence this antigen-presenting complex. Since one of the major components of the antigen-presenting microenvironment in these neonatal pigs is the interaction between CD16^+^ MHCII^+^ DC and stromal, endothelial MIL11^+^MHCII^+^ cells, we have used multiple-color fluorescence histology to address the question of the influences of early life rearing environment on development: alternative approaches such as flow cytometry or gene expression may provide greater depth of data but eliminate significant amounts of spatial information.

Pigs are valuable, tractable, preclinical models for humans (38) since they share many features of gastrointestinal physiology, immunology, microbiology, diet, and pathologies (39–42). In addition, precocial piglets are important models for studies of the impact of early environment on physiological development since their self-sufficiency permits early separation from their mothers, thus limiting the maternal influence at this critical period of developmental plasticity. Given the evidence for the influence of early-life environments on the later development of immunological disorders, we hypothesized that rearing piglets under different conditions during this early period of intense immune development would impact on the initial presentation of antigen to the immune system. Again, in our previous studies, effects of early life environment are initially observed in mucosal APCs and then subsequently on mucosal T-cells (31, 43). We further hypothesized that effects on antigen presentation would then affect the functional development of mucosal immunity, specifically by changing numbers of T_regs_ and, consequently, the potential for local immune regulation. By rearing piglets in either intensive (indoor) or extensive (outdoor) low-hygiene farm environments while their siblings were reared in a high-hygiene isolator facility from 1 day old, this study determined how the environment during the immediate postnatal period, and also over the first 8 weeks of life, influenced antigen presentation at locations within the gut mucosa where we have previously demonstrated the effects of weaning. It then assessed subsequent T_reg_ differentiation and development specific to the lamina propria of the distal portion of the porcine jejunum. Finally, it explored the effects of the consumption of a twice daily, pulse-dose cocktail of antibiotics against these divergent neonatal backgrounds both in terms of antigen presentation and the development of mucosal T_reg_ populations.

## Materials and Methods

### Animal Model

**Figure 1:**
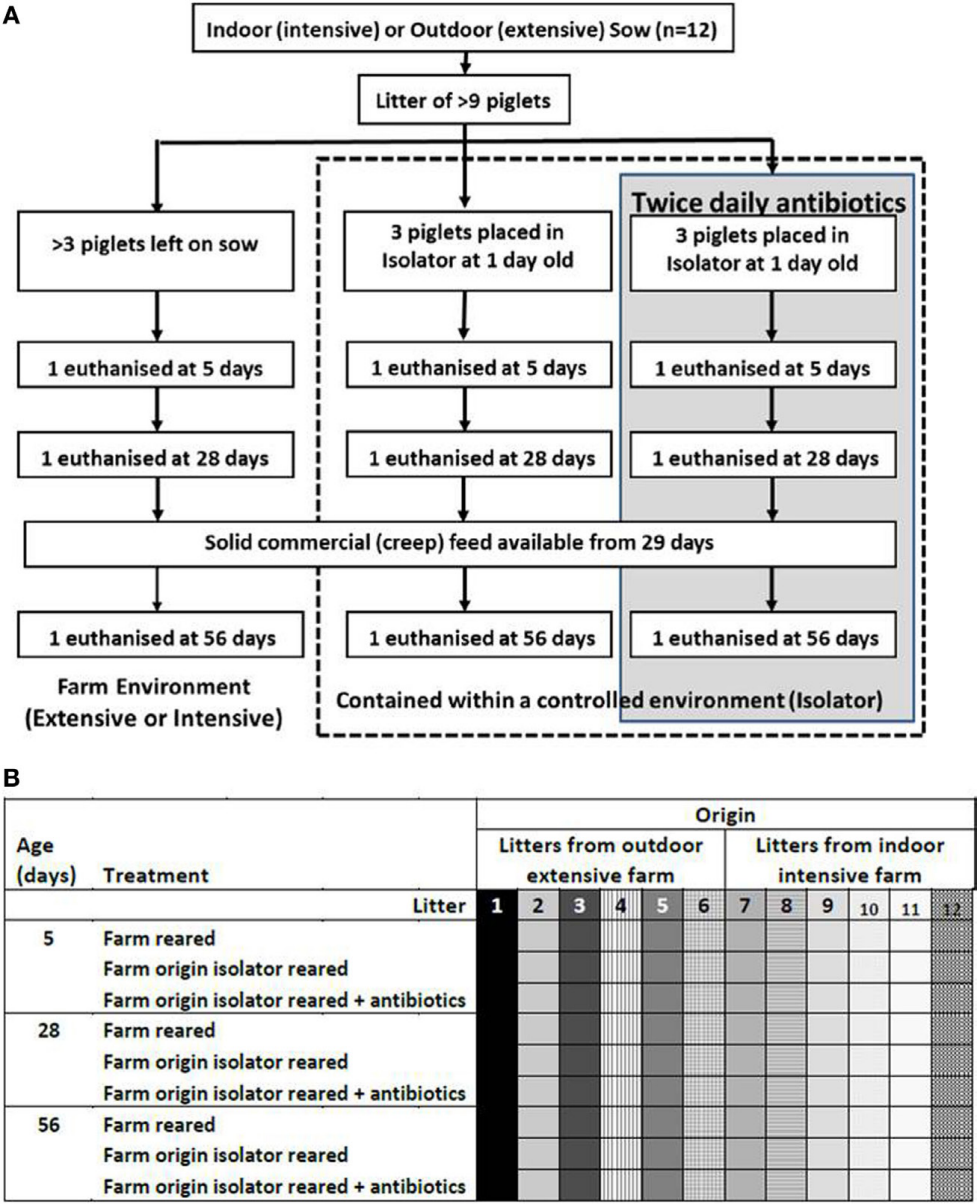
Animal model. Piglets were either left with their mothers or individually housed in a high-hygiene isolator facility from 1 day old **(A)**. Half the piglets contained within the isolator received twice-daily oral doses of antibiotics from 1 day old throughout the duration of the study (56 days). Piglets were not fully weaned, but did receive solid (creep) feed from 29 days. Outdoor extensive and indoor intensive trials were run concurrently and this was repeated six times to give *n* = 6 litter-matched piglets/group/timepoint (108 piglets in total from 12 sows). The six treatment groups were outdoor extensive farm-reared, outdoor extensive farm-origin isolator reared; outdoor extensive farm-origin isolFator-reared with antibiotics, indoor intensive farm-reared, indoor intensive farm-origin isolator reared, indoor intensive farm-origin isolator reared with antibiotics. Litters are depicted by shading and individual piglets a represented by each square **(B)**. Each litter was represented by one piglet in each treatment group and at each timepoint (5, 28, and 56 days).

Animal housing and experimental procedures were all performed at the University of Bristol Veterinary Science School in accordance with local ethical guidelines: all experiments were approved by the Bristol Animal Welfare and Ethical Review Body (AWERB) and were performed under a UK Home Office License. To examine the effects of neonatal and rearing environments, 108 piglets from 12 litters (6 litters from landrace × large white F1 hybrid sows on an intensive indoor farm and 6 litters from landrace × large white F1 hybrid sows on an extensive outdoor farm) were allocated into six equal groups of 18 piglets approximately 24 h after birth: three groups of piglets from the intensive, indoor farm and three from the extensive, outdoor farm. Litters were stratified through the experimental groups to ensure each litter was represented within each group. The farms were chosen as they used sows originally from the same genetic source rather than breeds appropriate for indoor intensive and outdoor extensive rearing. All sows had been artificially inseminated with semen from the same Hylean Large White boar to further limit genetic variation. To provide a high-hygiene rearing environment, two groups of indoor, intensive-origin pigs and two of outdoor, extensive-origin piglets were moved to an SPF facility maintained under positive-pressure with HEPA-filtered air and disinfected in Virkon^®^ and fumigated with formaldehyde gas (Alphagen Prills pellets, Antec AH Int., Sudbury, UK) prior to receiving piglets. Piglets in the high-hygiene SPF facility were individually housed on slatted flooring and automatically fed hourly with bovine milk-formula (Piggimilk, Parnutt Feeds, UK) similar to that fed to human infants. Two of these high-hygiene piglet groups (one of indoor intensive origin and one of outdoor extensive origin, *n* = 18 each) received a twice-daily antibiotic cocktail of Baytril (Bayer Healthcare, Uxbridge, UK) at a concentration of 5 mg enrofloxacin/kg body weigh and Amoxinsol 50 at a concentration of 10 mg amoxicillin trihydrate/kg bodyweight (Vétoquinol, UK Ltd., Buckingham, UK) given orally for the duration of the study. The remaining, litter-matched siblings (two groups) were left on their respective farm (indoor intensive or outdoor extensive) and were nursed by their mothers until 56 days (low-hygiene). Indoor litters remained in the farrowing unit for 28 days before being moved, with their sows, to larger indoor pens and provided with straw bedding, while the outdoor litters were free-range on grass with access to their sows and to farrowing arcs at night for the duration of the study. All piglets received standard husbandry iron injections within hours of birth, and also intramuscular multivitamins at 2-week intervals to compensate for the vitamin deficiency which may occur with prolonged antibiotic usage. To control for the effects of vitamins, multivitamins were given to all piglet groups, not just those receiving antibiotics, and none of the piglets were vaccinated. Additional solid feed (Multiwean, SCA Nutrition Ltd., Thirsk, UK) was available to all piglets from 29 days (*ad libitum*). Piglets were euthanized by an injection of sodium pentobarbitone (Euthesate, Willows Francis Veterinary Ltd., Crawley, UK) at 5, 28, and 56 days after birth and samples collected for analysis. The model resulted in *n* = 6 litter-matched piglets/group/timepoint. Groups were indoor intensive farm-reared; indoor intensive-origin isolator reared; indoor intensive-origin isolator-reared plus antibiotics; outdoor extensive farm-reared; outdoor extensive-origin isolator reared; outdoor extensive-origin isolator-reared plus antibiotics. Figure 1 is a schematic of the animal model. All piglets remained healthy throughout the trial and sows did not receive antibiotics either during pregnancy or beyond.

### Fluorescence Immunohistology

In our previous studies, we have identified changes in immune cell types associated with age and with treatments, particularly associated with environmental and dietary manipulation, in proximal and distal jejunum (44). Specifically, gene expression analysis has identified upregulation of genes associated with MHC class I and II processing pathways at these sites (13). Proximal and distal jejunum (avoiding Peyer’s patches) was identified in each piglet at 25 and 75%, respectively along the length of the small intestine, which permitted consistency in the location of the tissue sample collected between piglets. The tissues were snap frozen then serial, 5 µm sections were cut using a Model OTF cryotome (Brights Instrument Company Ltd., Huntingdon, UK). Sections were air dried for 24 h then fixed by immersion in acetone for 15 min. Slides were dried prior to storage at −80°C. A freezer malfunction resulted in loss of day 56 indoor isolator tissue samples before T_reg_ analysis could be completed. Thus, these data are missing from Figure 6.

Non-specific binding sites were blocked using 10% goat and pig serum and the samples were then stained with monoclonal antibodies. For the analysis of antigen presentation, the following monoclonal antibodies were used: anti-porcine CD14 (clone MIL2), anti-porcine CD16 (clone G7), anti-porcine intestinal capillary endothelium (clone MIL11), and anti-porcine MHC class II DR (clone MSA-3). Binding was detected with the following: goat anti-mouse IgG_2b_ TRITC (Southern Biotechnology), goat anti-mouse IgG_1_ FITC (Southern Biotechnology), biotinylated goat anti-mouse IgE (Southern Biotechnology) detected with AMCA-Avidin D (Vector Laboratories), and goat anti-mouse IgG_2a_ AlexaFluor 633 (Invitrogen). For the analysis of Foxp3^+^ T-cell numbers, the following monoclonal antibodies were used: anti-porcine CD4 (clone MIL17), anti-mouse Foxp3 (clone FJK-16s), and anti-porcine CD25 (clone K231.3B2). Binding was detected with the following: goat anti-mouse IgG_2b_ TRITC (Southern Biotechnology), goat anti-rat IgG FITC (Stratech), and biotinylated goat anti-mouse IgG_1_ (Southern Biotechnology), detected with AMCA-Avidin D (Vector Laboratories).

### Image Analysis

Image capture and proportional area of CD14, CD16, MIL11, and MHCII, or for CD4 and CD25 staining were analyzed using an in-house macro and ImageJ version 1.44 (45). Briefly, 10, 16 bit grayscale images were captured for each piglet and each location along the small intestine (resulting in 60 representational images for each treatment group/site/timepoint) using a Leica DMR-B fluorescence microscope fitted with appropriate fluorescence filters. The proportion of positive pixels in each color channel was measured using a specifically developed in-house macro. This allowed quantification of positive staining (and co-staining) by the primary antibodies as previously described (43). Briefly, images were thresholded to positive or negative pixels for each fluorochrome independently. The number of pixels positive for each possible combination of fluorochromes was counted and expressed as a proportion of the total pixels within a specific area. Where distribution of proportions was skewed, a log transformation was applied to achieve a normal distribution and results expressed as Log_10_ (proportion of area). Because expression of Foxp3 was nuclear, numbers of CD4^+^CD25^+^Foxp3^+^ were analyzed using the ImageJ cell counter plugin. The density of CD4^+^ staining was such that it was not possible to determine cell numbers; instead the proportion of area which stained positive for CD4 was measured and the values, again, logged to achieve normal distributions (45). Ratios of T_reg_ to effector populations was achieved by dividing the total number of CD4^+^Foxp3^+^ T_regs_/mm^2^ of lamina propria by the proportion of pixels staining positive for CD4 within the exact same locations.

### Statistical Analysis

Multivariable linear regression was carried out on the four-color quantitative immunofluorescence data using piglet as the experimental unit and litter, tissue, age, origin, and rearing environment as variables. Individual differences between treatment groups were determined by least-significant differences as in our previous experiments (26). Initially, the proportion of pixels positive for MHCII, CD16, CD14, and MIL11 (endothelium) were analyzed as individual, dependant variables. However, the data also included co-localization of combinations of all four molecules, a total of 16 possible combinations representing co-expression by individual cells or by cells interacting with each other in tissues (31, 37). Such combinations are partially cross-correlated (data not shown): increases in CD16 associated with MHCII, for example, are associated with increases in CD16 alone or MHCII alone. In order to eliminate correlation between these 16 variables and to reduce the total number of dependant variables, principal component analysis (PCA) was carried out using correlation matrices (for variables of different scales). PCA was carried out using IBM SPSS statistics (IBM, Chicago, IL, USA) using the proportion of pixels positive and negative for all combinations of CD14, CD16, MIL11, and MHCIIDR as variables to explore antigen presentation within the intestinal mucosa. Similar analysis has been used on flow cytometry data to describe subsets of circulating pig monocytes and DCs, and has identified CD14^+^ monocytes, and CD1^−^ and CD1^+^ DCs (36).

## Results

### The Early-Life Environment Exerts a Complex Effect on the Expression of APC-Associated Molecules in the Intestinal Mucosa

**Figure 2:**
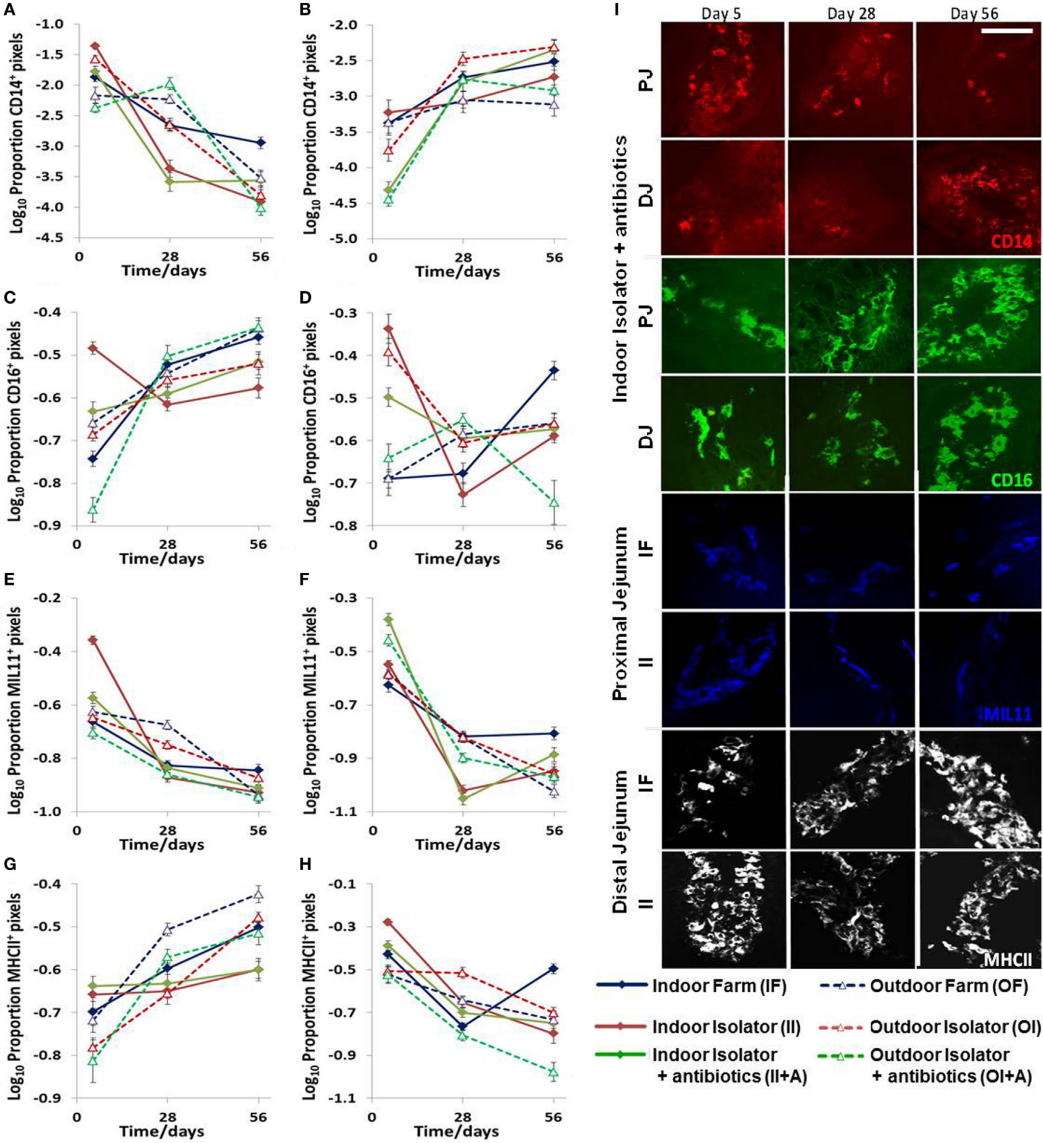
Effects of neonatal and early-life environments on expression of antigen-presenting cell-associated molecules in the intestinal mucosa. Quantitative analysis of proportion positive pixels by fluorescence immunohistology of proximal **(A,C,E,G)** and distal **(B,D,F,H)** jejunal lamina propria at 5, 28, and 56 days old in the following treatment groups (*n* = 6/treatment/timepoint): outdoor extensive farm reared; outdoor extensive origin, isolator reared; outdoor extensive origin, isolator reared with antibiotics; indoor intensive farm-reared; indoor intensive origin isolator reared; indoor intensive-origin isolator reared with antibiotics (108 piglets in total). Area staining positive for CD14 **(A,B)**, CD16 (intestinal dendritic cells) **(C,D)**, MIL11 (capillary endothelium) **(E,F)**, and MHC class II DR **(G,H)**. A table of significance for these data is available in Table S1 in Supplementary Material. Representational images **(I)** show positive staining for CD14 (red), CD16 (green), MIL11 (blue), and major histocompatibility complex class II (MHCII DR, white) within the intestinal mucosa at the specified sites in piglets from the groups indicated at 5, 28, and 56 days. Scale bar = 100 μm.

Statistically significant changes occurred in the expression of CD14, CD16, intestinal epithelium (MIL11), and MHCIIDR over time, by location along the jejunum, and in response to the treatment: farm of origin (indoor intensive or outdoor extensive and isolator reared), rearing environment (indoor intensive or outdoor extensive farm), or antibiotic supplementation against either (indoor intensive farm or outdoor extensive farm origin backgrounds) (*n* = 6 for each group, *p* < 0.0001 for all molecules; Figure 2; Table 1). However, the changes were complex and there were highly significant interactions between farm of origin, rearing environment, age, and site. For example, expression of CD14 followed a clear pattern: total expression in the proximal intestine decreased over time in all treatment groups (Figure 2A), while expression was upregulated over the same period in the distal jejunum, where the greatest difference between treatment groups occurred at 5 days (Figure 2B). By contrast, the picture was less clear for the porcine intestinal DC-associated protein CD16: although treatment effects were present, they were confounded by differences between age and site and difficult to interpret (Figures 2C,D; Table 1). The proportion of the area of lamina propria staining positive for intestinal epithelium (MIL11) was consistently greatest at 5 days compared to the 28 and 56 day timepoint in all treatment groups in both the proximal (Figure 2E) and distal (Figure 2F) jejunum. Overall, expression of MHCII tended to follow the inverse pattern to expression of CD14: that is, to increase over time in the proximal intestine and decrease in the distal intestine, with some exceptions (e.g., indoor farm at 56 days). This suggests that the two molecules (CD14 and MHCII) are likely to be on cell types responding differently to changes in the intestinal microenvironment.

**Table 1:** Table of variance for the expression of antigen presentation-associated molecules in the lamina propra of the porcine jejunum.

Test between-subjects effects	Log CD14	Log CD16	Log endothelium	Log MHCIIDR	PC1	PC2	PC3	PC4
Corrected model	**<0.0001**	**<0.0001**	**<0.0001**	**<0.0001**	**<0.0001**	**<0.0001**	**<0.0001**	**<0.0001**
Site	**<0.0001**	0.4426	0.0158	**<0.0001**	**<0.0001**	0.0001	0.0254	0.2974
Age	**<0.0001**	0.1424	**<0.0001**	**<0.0001**	0.0051	**<0.0001**	**<0.0001**	**<0.0001**
Origin (extensive/intensive)	0.1170	0.7726	0.2206	**<0.0001**	0.0650	0.0055	**<0.0001**	0.6725
Rearing (extensive/intensive; isolator; isolator + antibiotics)	0.0748	0.0007	0.3497	**<0.0001**	**<0.0001**	**<0.0001**	**<0.0001**	**<0.0001**
Age*Origin	**<0.0001**	**<0.0001**	**<0.0001**	**<0.0001**	**<0.0001**	0.0217	**<0.0001**	0.0008
Age*Rearing	**<0.0001**	**<0.0001**	**<0.0001**	**<0.0001**	0.0026	**<0.0001**	**<0.0001**	**<0.0001**
Site*Age	**<0.0001**	**<0.0001**	**<0.0001**	**<0.0001**	**<0.0001**	**<0.0001**	**<0.0001**	**<0.0001**
Origin*Rearing	0.0002	0.0041	0.1740	0.0159	**<0.0001**	0.6506	0.0082	0.1468
Site*Origin	0.0066	0.3920	0.3172	**0.0000**	0.7096	0.0126	0.0004	**0.0001**
Site*Rearing cat	0.0035	0.0004	**<0.0001**	**<0.0001**	0.0728	0.1537	**<0.0001**	**<0.0001**
Age*Origin*Rearing	**<0.0001**	**<0.0001**	**<0.0001**	**<0.0001**	0.0012	**<0.0001**	**<0.0001**	0.0060
Site*Age*Origin	**<0.0001**	**<0.0001**	**0.0001**	**<0.0001**	0.0013	0.6935	**<0.0001**	0.0008
Site*Age*Rearing	**<0.0001**	**<0.0001**	**<0.0001**	**<0.0001**	**<0.0001**	**<0.0001**	0.0084	**<0.0001**
Site*Origin*Rearing	0.1000	0.0056	**<0.0001**	0.0371	0.1532	0.0698	0.0127	0.0005
Site*Age*Origin*rearing	0.0001	0.0060	0.0091	**0.0000**	0.0010	0.0005	**<0.0001**	**<0.0001**

*Total staining of molecules identified by fluorescence immunohistology using CD14, CD16, MIL11 and MHCII DR, and all combinations as factors (16 in total). During principal component analysis, variables were: site—proximal or distal jejunum; age—5, 28, or 56 days old; origin—born on either indoor or outdoor farm; treatment outdoor extensive farm reared; outdoor extensive farm-origin isolator reared; outdoor extensive farm-origin isolator reared with antibiotics; indoor intensive farm-reared; indoor intensive farm-origin isolator reared; indoor intensive farm-origin isolator reared with antibiotics. Gray background indicates significant values showing that Factors 2 and 4 were associated with treatment, but not the farm or origin (indoor intensive or outdoor extensive), whereas Factor 3 was associated with farm origin, but not treatment (rearing conditions)*.

*Shading indicates those values <0.0001*.

### Early Life Environment Affects Co-Expression of Molecules Associated With APC Function

**Figure 3:**
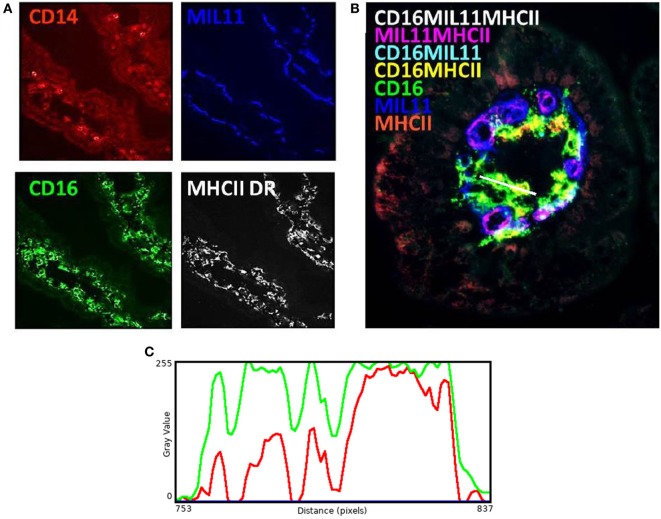
Antigen presentation within the intestinal lamina propria is complex. Representation of fluorescence immunohistology from the distal jejunum lamina propria of a 28-day-old isolator-reared piglet. **(A)** CD14 (red), CD16 (intestinal dendritic cells) (green), MIL11 (capillary endothelium) (blue), and MHCII DR (white). **(B)** combined image of antigen presentation-associated molecule s CD16 (green), MIL11 (blue), MHCII (red), CD16 and MIL11 co-staining (cyan), CD16 and MHCII co-staining (yellow), MIL11 and MHCII DR co-staining (magenta), and CD16, MIL11, and MHCII DR co-staining (white). **(C)** Color profile of tissue indicated by the white bar in **(B)** showing red (MHCII) and green (CD16).

**Figure 4:**
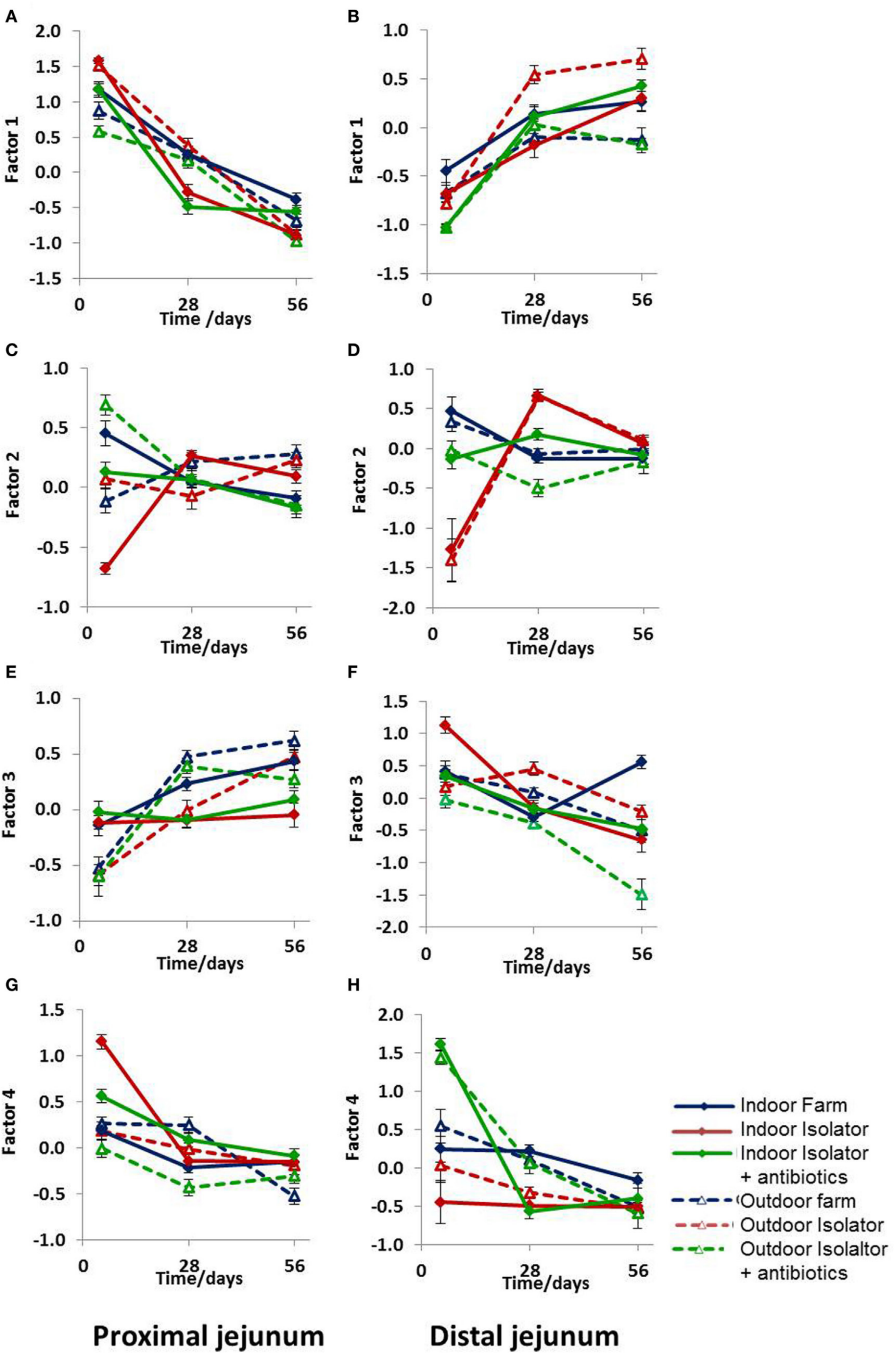
Principal component analysis (PCA) of antigen presentation data. PCA of antigen presentation-associated molecules identified by fluorescence immunohistology using CD14, CD16, MIL11, and MHCII DR positive staining and all combinations as factors (16 in total) at 5, 28, and 56 days old. **(A,B)** Factor 1, **(C,D)** Factor 2, **(E,F)** Factor 3, and **(G,H)** Factor 4. **(A,C,E,G)** Proximal jejunum and **(B,D,F,H)** distal jejunum. Treatment groups: outdoor extensive farm reared; outdoor extensive farm-origin isolator reared; outdoor extensive farm-origin isolator reared with antibiotics; indoor intensive farm-reared; indoor intensive farm-origin isolator reared; indoor intensive farm-origin isolator reared with antibiotics. A table of significance for these data is available in Table S2 in Supplementary Material. Error bars = SEM, *n* = 6/treatment group/timepoint.

**Figure 5:**
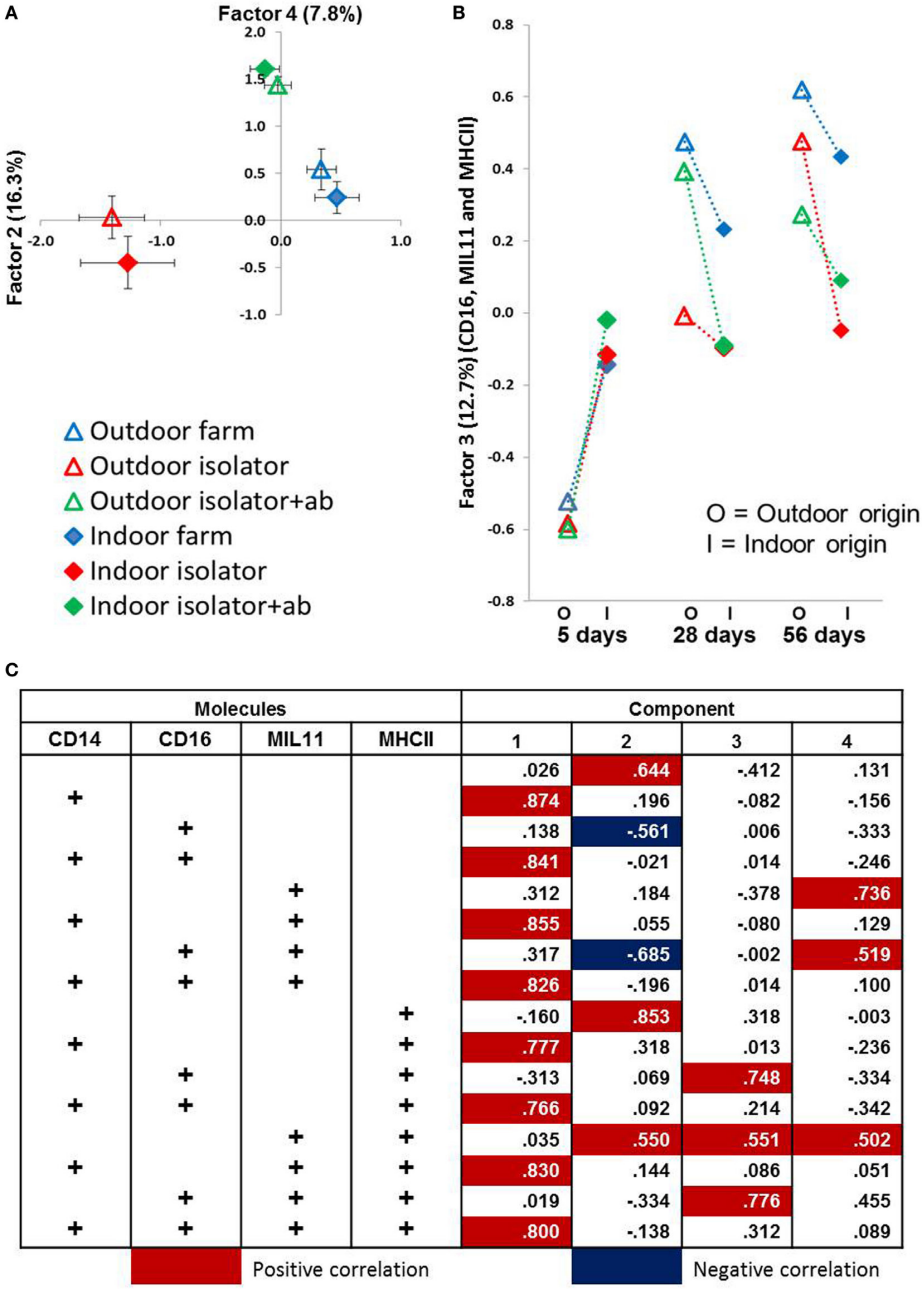
Principal component analysis (PCA) identifies significant factors associated with either origin of birth or rearing treatment. Antigen presentation-associated molecules identified by fluorescence immunohistology of intestinal lamina propria (using CD14, CD16, MIL11 and MHCII DR positive staining, and all combinations as factors) used in a PCA. **(A)** Factor 2 (*X*-axis) plotted against Factor 4 (*Y*-axis) from distal jejunum of 5-day-old piglets. Each plot represents a treatment group (*n* = 6). Factor 2 explains 16.3% variance of the data while Factor 4 explains 11.3%. Error bars = SEM. **(B)** Factor 3 from PCA using data obtained from fluorescence histology (using the factors above) obtained from analysis of the lamina propria of the proximal jejunum. Plots represent treatment groups at 5, 28, and 56 days. O = outdoor-associated animals (outdoor extensive origin, reared, or antibiotic treatment), while I = indoor intensive-associated animals (origin, rearing or with antibiotic treatments). Factor 3 explains 12.8% of the variation observed. Error Bars = SEM, *n* = 6/treatment group/timepoint. **(C)** Loadings from PCA results shown in **(A,B)** in relation to total positive staining for each of CD14, CD16, MIL11 (capillary endothelium), and MHCII.

While these results are complex and difficult to interpret, they clearly demonstrate that differing neonatal environments, rearing conditions, and oral antibiotics during early-life impact on the level of expression of molecules associated with antigen presentation in both the proximal and distal jejunum of young piglets, and that some of these effects appear to be sustained (Figure 2). However, total expression of CD14, CD16, MIL11, and MHCII represents the simplest possible analysis of the immunofluorescence data. In the real images, co-expression of different sets of molecules is apparent (Figure 3). In addition, Figure 3C demonstrates the problem of visual interpretation of multiple-color immunohistology: although many cells appear “green only,” they are, in fact, both red (MHCII) and green (CD16) as the color profile demonstrates. We have previously described analysis of co-expression of sets of four molecules as a way of understanding cellular interactions in tissues (31, 45). In order to simplify the analysis of 16 possible combinations of expression of four molecules, all of which may be partially correlated, we carried out PCA to identify a smaller set of four latent variables to be analyzed by multivariable linear regression. Principal components 1–4 explained 76% of the total variation in the original data. Figure 4 shows changes in magnitude of these four principal components, which now represent co-localization as well as total expression, over time, and between groups. Loadings associated with these components are shown in Figure 5C.

All four principal components were, again, significantly associated with farm of origin, rearing environment, site, and age. However, associations between specific principal components and farm of origin or site were easier to interpret than the original, unidimensional data (total expression of CD14, CD16, MIL11, and MHCII). Loadings on principal component 1 demonstrates that it corresponds almost completely to expression of CD14 and CD14 is not represented in loadings for any of the other factors, suggesting that it is not linked to expression of CD16, MHCII or the endothelial antigen MIL11. Studies on pig blood DCs have suggested that CD14 expression is actually confined to monocytes, and the lack of association between CD14 in this study and the other molecules associated with pig DCs is consistent with this (36).

CD14 is a good example of a molecule where the PCA analysis demonstrates a lack of co-localization: PCA Factor 1 is determined solely by the presence or absence of CD14, not by the presence or absence of any other molecule. By contrast, PC Factor 3 is positively associated with the presence of CD16 and MHCII (but not either alone), MIL11 and MHCII (but not alone), or all three together (CD16, MIL11, and MHCII). Since CD16 and MIL11 are expressed on different cell types, the fact that the level of co-localization is influenced by farm of origin or rearing environment indicates that the interaction is influenced by these treatments. Principal components 2, 3, and 4 were highly significantly associated with farm of origin (first 24 h) or rearing environments (Table 1; *p* < 0.0001). Together, principal components 2 and 4 clearly separate the three rearing environments (farm isolator, isolator plus antibiotics) at 5 days old (Figure 5A). Examination of the loadings (Figure 5C) suggests that these two principal components are positively associated with MHCII alone or in association with MIL11 and do not describe antigen-presenting function associated with MHCII^+^CD16^+^ DCs, that is, they primarily describe presentation by endothelial cells. Principal component 3 was highly significantly associated with farm of origin [Table 1; *p* < 0.0001 for farm of origin (origin), age*origin and site*age*origin]. The interaction between farm of origin and age is shown in Figure 5B, where piglets from the indoor, intensive farm had higher levels of PC3 than those from the outdoor, extensive farm at 5 days (regardless of rearing environment from 1 day old), but lower levels at 28 and 56 days old. This reversal occurred because of an increase in PC3 in outdoor-origin piglets between 5 and 28 days. However, it should be noted that, since piglets are born with very little intestinal MHCII (46), the presence of appreciable levels of PCA Factor 3 at 5 days in these piglets is likely to indicate an increase earlier than day 5. Importantly, lower levels of PC3 were apparent at 56 days not only in piglets still on their respective farm of origin but also in piglets removed from the farm of origin at 1 day old and transferred to a constant rearing environment (isolator). That is, PC3 remained significantly different in piglets from the indoor intensive and outdoor extensive farms 55 days after they were all moved to the isolator environment, suggesting that events either in pregnancy or the first day of life had a sustained impact on the development of the antigen-presenting architecture in the intestine. Examination of the loadings (Figure 5C) suggests that this principal component describes antigen-presenting function associated with MHCII^+^CD16^+^ DCs and with the previously described interaction with MHCII^+^MIL11^+^ capillary endothelial cells (31).

### Neonatal and Rearing Environments, and Treatment With Antibiotics, Are Associated With Significant Changes in CD4^+^ T-Cell Area, and in the Proportion of T_regs_ in the Intestinal Mucosa

**Figure 6:**
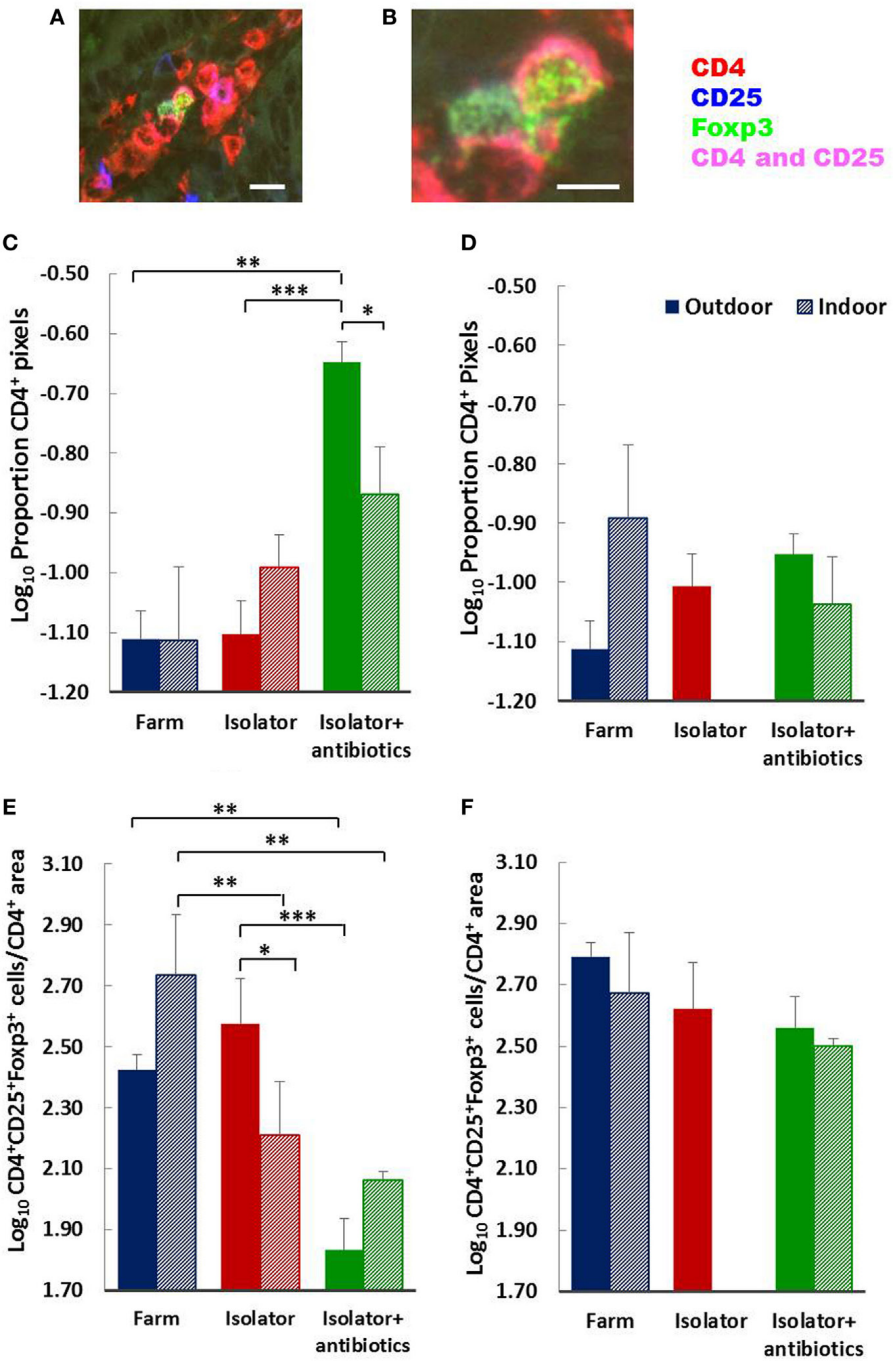
Birth environment, rearing environment, and treatment with antibiotics affects CD4^+^ T-cell area, and the ratio of CD4^+^Foxp3^+^ regulatory T-cells (T_regs_) to CD4^+^ T-cell area. Representational images of CD4^+^CD25^+^Foxp3^+^ T_regs_ in the lamina propria of a 28-day-old piglet. Where CD4 (red) and CD25 (blue) are co-expressed on the cell surface magenta is observed. The transcription factor Foxp3 (green) is localized to the nucleus [**(A)** enlarged image in **(B)**]. The proportion of CD4^+^ staining **(C,D)** and T_reg_ (CD4^+^CD25^+^Foxp3^+^) cell numbers as a proportion of CD4^+^ staining **(E,F)** were quantified using fluorescence immunohistology in the lamina propria of the distal jejunum when piglets were 28 **(C,E)** and 56 **(D,F)** days old. The log ratio was obtained by dividing the number of CD4^+^CD25^+^ Foxp3^+^ T-cells in each of 60 images/treatment/timepoint by the proportion of the area staining positive for CD4 in each of the same areas assessed (regions of interest). The CD4^+^ area was too dense to permit single cell enumeration. Solid columns represent piglet groups which originated and were reared on the outdoor extensive farm (blue), originated on the outdoor extensive farm and were then reared in the isolator from 1 day old (red), or originated on the outdoor extensive farm and then reared in the isolator and supplemented with antibiotics from 1 day old and throughout the trial (green). Shaded columns represent piglets which originated and were reared on the indoor intensive farm (blue), originated on the indoor intensive farm and then reared in the isolator (red), or originated on the indoor intensive farm then reared in the isolator and supplemented with antibiotics (green). Data for the indoor isolator piglets at 56 days old was not available. Error bars = SEM, *p ≤ 0.05, **p ≤ 0.001, and ***p ≤ 0.0001. Scale bar = 10 µm in **(A)** and 5 µm in **(B)**.

Total numbers (area) of CD4 T-cells were largely unaffected by treatment (origin or rearing) with the single exception of an increase in the isolator piglets from an outdoor environment given antibiotics (Figures 6C,D). The status of the farm (intensive or extensive) had no significant impact on the proportion of mucosal T-cells with T_reg_ phenotype at either 28 of 56 days for those piglets which were reared on their respective farm and sucked by their mothers. However, removing indoor, intensive, farm-origin piglets from their mothers at 1 day old and rearing them in a high-hygiene isolator facility caused a significant reduction in the proportion of T_reg_ at 28 days compared their farm-reared siblings (*p* < 0.001), indicating a change in the ratio of regulatory to effector T-cells (Figure 6E). The administration of oral antibiotics to these piglets had no further impact on this reduction in T_regs_ compared to farm-reared siblings. By contrast, the proportion of T_regs_ in the intestinal mucosa remained unaltered in those piglets originating from the outdoor, extensive farm which were relocated to the same isolator facility at the same time. However, in these outdoor-origin piglets, antibiotic administered from 1 day old, once in the isolator, was linked to a significant reduction in the proportion of T_regs_ compared to their outdoor-origin, isolator-reared counterparts which had not received antibiotics (*p* < 0.0001). Consistent with an effect of isolator-rearing alone in indoor intensive-origin but not outdoor extensive-origin piglets, there was a significant difference in the proportion of T_regs_ present in intestinal mucosa between these groups (*p* = 0.02), suggesting that differences driven by the respective farm environments was sustained for at least 28 days following isolator rearing in individual cages. This effect of farm of origin or rearing environment appeared transient, since there were no observable differences in the proportion of T_regs_ within the total CD4 T-cell population between any further treatment groups of 56-day-old piglets (Figure 6F).

## Discussion

There has been an increase in the incidence of allergic and autoimmune disease in western societies over the last 50 years and the rapidity of this escalation over such a limited genetic interval implicates environmental influences (47). One possible explanation which has been proposed is the “hygiene hypothesis” (48) in which decreased exposure to intracellular infections such as measles and tuberculosis was inversely correlated with allergy (49). The hypothesis has undergone various modifications to date and several recent studies have directly linked the composition of the gut microbiota of neonates to the later development of allergy (10, 50). Here, we present direct experimental evidence that perinatal environment, including the first day of life and the rearing conditions to 56 days after birth, affects local regulatory and antigen presentation potential in the gut mucosa. Furthermore, the impact of antibiotics throughout this timeframe on the development of the immune system was highly dependent on environmental conditions during the perinatal period, suggesting that multiple factors interact to trigger long-term changes in immune development. This is consistent with the idea that early life programming events may alter developmental physiology and predispose to later immunological disease. It is also consistent with the observation that the protective effects of farm environments are not universal, appear highly dependent on the type of farming practiced and on the subsequent rearing environment, and are also restricted to exposure during childhood. Thus, farmers have significantly increased morbidity and mortality from non IgE-mediated respiratory disease than the general population, despite reduced tobacco use, probably linked to prolonged exposure to large, confined animal feeding operations, and intensive practices (51). In addition, delaying exposures to farm environments until adulthood can lead to increased allergic sensitization (52), as can early farm exposure by children living in low-income countries (53).

A primary reason for the paucity of mechanistic knowledge linking farm environments to protection against the development of allergies is the difficulties involved in establishing useful animal models which directly compare farm-exposed animals to non-farm counterparts with similar genetic backgrounds. This is especially difficult during early life when rodents, for example, are vulnerable and highly dependent on their mothers. However, removing domesticated farm animals to clean environments is clearly a more practical approach, especially during early-life since the offspring of such species are generally more robust and less reliant on maternal care than rodent counterparts. In addition, specific farm species can be valuable models for humans with regard to relative physiology. In those studies which have been performed in the laboratory and/or in rodents, emulating the complex antigenic load provided by farm environments is often achieved by administering LPS, which could be considered a proinflammatory challenge (54, 55). These studies have provided some mechanistic insight into immune development and subsequent allergy protection, especially in regard to asthma. However, whether such stimuli reflect a farm in terms of the unique factors, microbial-derived and otherwise, which it provides is unclear. Farm dust also contains bacterial compounds other than LPS as well as plant and animal-derived antigens and the specific composition will be highly dependent on the type of farm from which it was derived. However, in one study, farm dust was demonstrated to reduce HDM-induced asthma when given prophylactically to mice, but this study did not assess immunological changes under such conditions (55), and data regarding changes in intestinal immunity directly induced by exposure to farm environments are rare.

Most epidemiological studies are unable to differentiate whether maternal exposure to specific farming environments during pregnancy, the neonatal period, or later rearing conditions (or indeed all three) elicits a protective effect since exposure, especially in humans, generally occurs throughout gestation and well into childhood. Although most studies consider the value of early farm exposure to be during the first year of life, several other reports have also identified beneficial effects of maternal exposure to farm environments during late pregnancy, and a continuing inverse association between atopic sensitization in school age children (56–58), suggesting that multiple early windows of opportunity may exist. By rearing half-sibling piglets with similar genetic backgrounds on either an indoor (intensive) or outdoor (extensive) farm for the first day of life before individually housing them in a high-hygiene isolator facility, we were able to determine that a combination of gestational conditions or the environment during the first day of life, had effects on T_reg_ to CD4^+^ effector area ratios by 28 days which were no longer apparent by 56 days. Under commercial rearing conditions, piglets begin to take solid food at around 2 weeks old and are weaned at 4 weeks old, and the transient effect on T_regs_ in the intestine, therefore, coincides with the maximum exposure to novel food antigens. Consistent with this, our previous studies have shown that such isolator-reared piglets do make more antibody to novel food proteins after weaning at 28 days than do their farm-reared siblings (26).

While effects of the first day and of subsequent rearing environments on subsequent development of T_regs_ were apparent at weaning but not later, effects on antigen-presenting microenvironments were sustained for the full period of the experiment. Principal component Factor 3 was reduced in extensive, outdoor-origin piglets and, therefore, appears to be negatively linked to development of mucosal T_regs_. However, additional Factors (1 and 2) together explained the variability in the model associated with rearing environment. Together, these findings suggest that two different immunological pathways associated with antigen presentation were being influenced by the perinatal environment or by the environment during infancy, respectively. Thus, the “windows” observed in human infants during gestation, the first year, and early school age are likely to involve different mechanisms, and interventions such as pro- or prebiotics may need to be targeted differently in the immediate postnatal period and during infancy. In our experiments, principal component 3 (associated with the farm of origin) was associated with the presence of CD16^+^MHCII^+^ DCs and their interactions with MHCII^+^ stromal cells, as previously described by our group (31). Principal components 2 and 4 were associated with stromal (endothelial) MHCII. Expression of stromal MHCII and antigen presentation by stromal cells, specifically enterocytes, also occurs in humans and mice (59–61), where it has been associated with antigenic load. It may be that these components identify increased involvement of stromal APCs as a consequence of specific forms of microbial exposure.

The effects of the addition of twice daily, pulse-doses of oral antibiotics with broad-spectrum bactericidal activities to isolator-reared piglets were highly dependent on the farm of origin (extensive/intensive). Isolator piglets originating from the intensive indoor farm already had reduced proportion of T_regs_ within CD4 T-cells and were not further affected by antibiotics. By contrast, the ability of early exposure to an outdoor, extensive environment to maintain the potential for immunoregulation in the intestine was abrogated by antibiotic treatment. There are two important implications from this result. First, that the factors associated with the outdoor, extensive-farm which affected the development of mucosal immune regulation were probably microbial in origin and linked to initial colonization of the gut from both the mothers and from the environment immediately following birth. Second, that the effects on predisposition to allergic disease of antibiotic administration to human infants are likely to be highly dependent on previous exposure to environmental microbiota during early life. This latter point may explain the discrepancies between some of the epidemiological studies and, in addition, suggests that specific groups, such as the Amish, may be more susceptible to the effects of antibiotic administration in early-life.

In conclusion, our results are consistent with there being at least two different windows of opportunity in which farm environments influence immune development in the intestinal mucosa; one before or during the immediate neonatal period and one during later infancy. Environmental influences during these crucial periods of developmental plasticity appear to include two alternative immunological developmental pathways involving antigen presentation by combinations of DC and alternative, non-professional APC populations. Our data suggest that exposure to extensive, but not intensive, farm environments during gestation and the first day of life is linked to improved potential for local immune regulation around weaning, even when it is followed by subsequent rearing in high-hygiene isolator facilities, but that the addition of antibiotics abrogated this. Our data further suggest that gestation and the first day of life provides the greatest opportunity for an extensive farm environment to improve regulatory potential, compared to the later rearing environment during infancy. We demonstrate that, mechanistically, differences in regulatory potential driven by different environments may be linked to antigen presentation *via* alternative APC populations directly in the gut mucosa, and the detrimental influence of antibiotics strongly suggests that the underlying driver is the acquisition of initial intestinal bacterial colonizers from mothers and/or the environment shortly after birth. Our work has important implications for at-risk infants which have been exposed to factors such as cesarean delivery, early antibiotics (maternal or direct), or delayed discharge from high-hygiene hospital environments. These results suggest that very early interventions, for example, probiotics, may improve prognosis for these infants with increased likelihood of developing allergic and other inflammatory conditions in later life, but they may need to be targeted during specific windows in the perinatal period or in infancy. However, further work is required in order to determine the exact extensive farm-associated early-life factors which are driving this increased potential for local immune regulation around weaning.

## Ethics Statement

Animal housing and experimental procedures were all performed at the University of Bristol Veterinary Science School in accordance with local ethical guidelines: all experiments were approved by the Bristol Animal Welfare and Ethical Review Body (AWERB) and were performed under a UK Home Office License.

## Author Contributions

MB, DK, JP, BG and CS conceived the idea; ML and RB performed the experiments; ML, MB, ZC, and RB collected and analyzed the data; IM, DK, MB, ML, and CS interpreted the data; ML wrote the manuscript; ZC provided the Treg data; ML, MB, CS, and JP provided expertise on the pig model and pig immunology; MB and ML were responsible for the overall direction of the paper; all authors reviewed the manuscript and approved the version to be published.

## Conflict of Interest Statement

The authors declare that the research was conducted in the absence of any commercial or financial relationships that could be construed as a potential conflict of interest.
