# Supplementary material for: Development of Immune Cells in the Intestinal Mucosa Can Be Affected by Intensive and Extensive Farm Environments, and Antibiotic Use
Source: Front Immunol. 2018 May 16;9:1061. doi: 10.3389/fimmu.2018.01061 (PMC5964130; doi:10.3389/fimmu.2018.01061)
Supplement: Supplementary file 1 [file Table_1.DOCX]

**Supplementary Table 1:** Table of significance for Figure 2 (a to h). *p*<0.05 in **bold**

**Supplementary Table 2:** Table of significance for Figure 4 (a to h). *p*<0.05 in **bold**
